# Neurologists’ perspectives of cannabis-based medicines: results from an all-Ireland survey

**DOI:** 10.1007/s11845-025-03880-0

**Published:** 2025-02-03

**Authors:** Michael Savio, Hugh Kearney, Eric J. Downer

**Affiliations:** 1 Discipline of Physiology, School of Medicine, Trinity Biomedical Sciences Institute, Trinity College Dublin, Dublin 2, Ireland; 2 MS Unit, Department of Neurology, St. James’s Hospital, Dublin, Ireland; 3 Academic Unit of Neurology, School of Medicine, Trinity College Dublin, Dublin 2, Ireland

**Keywords:** Cannabis, Cannabis-based therapeutics, Ireland, National survey, Neurology

## Abstract

**Background:**

Advancements continue to be made in the development of medicines containing components of the *Cannabis sativa* L. plant. Consultants can prescribe specific cannabis-based products for a restricted set of indications in Ireland, with neurologists being at the forefront of therapy. Much debate on the therapeutic potential/efficacy of such cannabis-based products exists.

**Aim:**

The objective of this study was to conduct a national survey to determine the perspectives/views of Irish neurologists regarding the use of cannabis-based medicines.

**Methods:**

An online anonymous survey was conducted to capture the perspectives and experiences of neurologists in Ireland regarding cannabis-based therapeutics.

**Results:**

Thirty-four neurologists completed the survey in full, with study participants rating their knowledge of cannabis-based medicines as average. Data presented herein indicate that there is a need for educational programmes on the cannabinoid system and cannabinoid-based medicines, and the findings indicate that neurologists are interested in the use of cannabinoid-based medicines in their practice. Study participants were more divided with regard to the clarity of the process for accessing cannabis-based medicines, and the consensus is that the application process is unclear. Approximately one-third of participants have made an application to access medicinal cannabis-based products on behalf of a patient.

**Conclusions:**

Data presented herein indicates that the majority of neurologists surveyed are aware of the current systems in place to access cannabis-based products for medicinal use in Ireland and that some engagement with these systems has taken place. A key finding is that educational programmes on the cannabinoid system and cannabis-based medicines are required.

## Introduction

Commonly known for its recreational use worldwide, the hemp plant *Cannabis sativa* L. (*C. sativa*) has a lesser-known therapeutic role in modern medicine. In healthcare, the derivatives of *C. sativa* are being increasingly used in the management of a variety of conditions, including chemotherapy-associated nausea, spasticity in people with multiple sclerosis (pwMS) and seizures in Dravet and Lennox-Gastaut syndromes [1–3]. In particular, much scientific and clinical research has focused on two key plant–derived (phyto) cannabinoids, cannabidiol (CBD) and Δ^9^-tetrahydrocannabinol (Δ^9^-THC). THC is a euphoric component of the plant, while CBD is a non-euphoric phytocannabinoid [4]. Therapeutics based on THC and CBD have received regulatory approval as authorised medicines, including Sativex® (nabiximol; a 1:1 THC:CBD oromucosal spray), Epidiolex® (a pure CBD oil solution), Marinol® (dronabinol; a synthetic THC tablet) and Cesamet® (nabilone; a synthetic analogue of THC tablet) [2, 5–8]. Sativex® and Cesamet® are authorised medicines in Ireland for patients with certain conditions who have failed to respond to approved/authorised existing medications, with Epidiolex® an approved investigational product [9]. This reflects their manufacture from standardised extracts from the plant or from a synthetic source. The discovery of the endocannabinoid system, a system of endogenous cannabinoids and G-protein-coupled cannabinoid receptors, has renewed scientific interest in the properties, biological effects and therapeutic value of components of *C. sativa* [10, 11].

Cannabis-based medicinal products are currently not available directly to the Irish marketplace [9, 12]. Since 2017, there have been some advances in the development of avenues to access cannabinoid-based products for medicinal purposes in the Republic of Ireland. Indeed, in 2017, the Health Products Regulatory Authority (HPRA) conducted a review of medical cannabis, recommending that such medicines should be available for a restricted set of conditions, namely severe MS-associated spasticity, severe, treatment-refractory epilepsy and intractable nausea/vomiting associated with chemotherapy [13]. Subsequently, in 2019 a Medical Cannabis Access Programme (MCAP) was formally established with a view to determining the effectiveness and safety of a range of unauthorised cannabis-based products while contributing clinical data to enable future decisions to be informed by a strong evidence base [14]. This system would operate alongside the Ministerial licence scheme, and under the MCAP, medical consultants can prescribe specific cannabis-based products for a restricted set of indications in Ireland. A number of controlled cannabis-based products (containing various concentrations of CBD and THC) are currently listed under the MCAP, including Aurora High CBD Oil Drops, CannEpil™, Tilray Oral Solution, Aurora Sedamen Softgels, Oleo Bedrobinol and Oleo Bedrocan [14].

Despite the growing use of cannabis-based medicines, the existing literature suggests that there is stigma related to the use of cannabis therapeutics [15], and a lack of knowledge in terms of cannabinoid biology, pharmacology and therapeutics [16, 17]. Indeed, studies have found that clinicians report a gap in their knowledge in terms of cannabinoid-based medicines, in addition to a lack of confidence in such medicines [17, 18]. There are also concerns regarding potential misuse of prescribed cannabis-based products [19] in addition to clinician concerns regarding the impact of cannabis on mental and physical health [20]. Clinicians have identified a need for more education regarding treating patients with cannabis-based medicines [17, 21], with evidence that the knowledge of healthcare professionals regarding cannabis medicines tends to be acquired from medical literature, in addition from self-directed learning, media reporting and from other clinicians [22]. Potentially biased information from secondary sources may not necessarily be reliable. There is also data to indicate that clinicians agree that education regarding cannabis should be included in medical school curricula [22].

Overall, evidence suggests that there is a lack of education and concern regarding the prescription of cannabis-based medicines [17, 19, 21]. We conducted a national survey to determine the views and perspectives of Irish neurologists regarding the use of cannabis-based medicines in their practice.

## Materials and methods

### Study design

An online national survey was conducted from March to July 2024 to capture the perspectives and experiences of neurologists in Ireland regarding cannabis-based therapeutics. Neurologists were contacted via the Irish Institute of Clinical Neuroscience (IICN), which was asked to distribute the survey to their members. Electronic invitations were sent to IICN members, with invitations including a survey summary, consent information and a link to access the online survey via Qualtrics. Qualtrics was selected to host the survey as it is supported by Trinity College Dublin and data are securely stored. The survey was fully anonymous. Informed consent was obtained from each participant and the study protocol received ethical approval from the School of Medicine Research Ethics Committee at Trinity College Dublin, Ireland.

### Questionnaire design

The survey content was developed based on a review of the literature and reviewed by the research team that included a neurologist, medical student and academic cannabinoid researcher and comprised 13 questions that included Likert-style and binomial (yes/no) questions. In addition, the survey collected data regarding participant demographics, current practice location, knowledge of cannabinoid medicines that are available and in development, knowledge of the endocannabinoid system and the MCAP/Ministerial licence schemes in Ireland. The survey also consisted of questions regarding the neurologist’s experience using the MCAP/Ministerial licence schemes in Ireland and their opinions on cannabinoid-based medicines. A series of 10-point Likert scale questions were used to assess experiences towards the available educational programmes for cannabis-based medicines, the evidence base to support the use of cannabis-based medicines for symptom management in certain conditions and the process for accessing medicines. A 10-point Likert scale was also used to determine the interest of study participants in the use of cannabis-based medicines in medical practice. Finally, there was an opportunity for open comments to be made as free text. The survey took an estimated 5 min to complete.

### Data analysis

The data from the questionnaires was anonymised with code identifiers, transferred to Microsoft Excel and analysed using GraphPad Prism (version 10). The data was exported to GraphPad Prism for descriptive analysis, and data were summarised as frequencies and percentages. Data were tested for normality using the Shapiro–Wilk test, and non-parametric testing was employed using the Mann–Whitney *U* test. The significance level was set at *p* < 0.05. Descriptive statistics (frequencies, percentages, mean, standard deviation (SD) and range) were computed for participants who completed the survey in full (*n* = 34). Percentages were calculated for the participants’ responses to the questionnaire. Likert scale data are expressed as mean ± SD. For non-normal data presented in graph format, data are presented as whisker plots with minimum/maximum values and interquartile range (IQR).

## Results

### Study cohort characteristics

The study participant characteristics are indicated in Table 1. A total of 34 neurologists completed the survey in full, of which 18 (53%) were consultants and 16 (47%) were non-consultant trainees. Ten participants did not complete the survey in full and were not included in the analysis. Over half of the participants indicated that their current region of practice is in Dublin (56%). Other locations of practice included Cork (6%), Galway (9%), Waterford (3%) and Northern Ireland (12%). Fifteen percent of the study cohort did not specify the region in which they currently practice.

**Table 1:** Participant characteristics

Characteristics	***n* (%)**
Profession	
Consultant neurologist	18 (52.9)
Trainee neurologist	16 (47.1)
Practice/employment location	
Dublin (*n*)	19 (55.9)
Cork (*n*)	2 (5.9)
Galway (*n*)	3 (8.8)
Waterford (*n*)	1 (2.9)
Northern Ireland (*n*)	4 (11.8)
Not specified (*n*)	5 (14.7)

Number of neurologists that completed the survey in full = 34

### Participants’ opinion of their knowledge of cannabis-based medicines

**Fig. 1:**
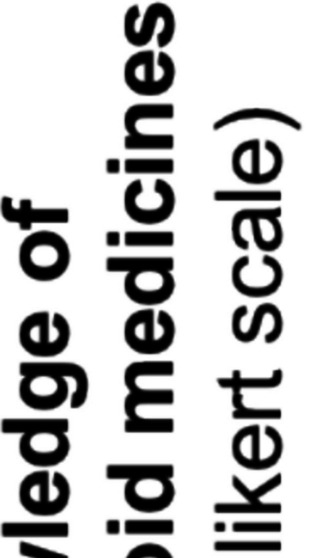
Consultants exhibit a significantly higher score than non-consultant trainees in terms of the opinion of their knowledge of cannabis-based medicines. All study participants were asked to rate their knowledge of cannabinoid medicines using a 5-point Likert scale. Results presented in the 5-point Likert scale are the following: 5 = excellent knowledge, 4 = good knowledge, 3 = average knowledge, 2 = poor knowledge and 1 = no knowledge. Data are presented as whisker plots with minimum/maximum values and IQR. Symbols indicate individual data points. Statistical analysis: Mann–Whitney *U* test. *p < 0.05 when comparing Likert scale scores between the consultant and trainee group

Participants were asked to rate their knowledge of cannabis-based medicines, and data were collected using a 5-point Likert scale where 1 is “no knowledge” and 5 is “excellent” knowledge. Data presented in Fig. 1 indicate that consultants exhibit a significantly higher score than non-consultant trainees in terms of the opinion of their knowledge of cannabis-based medicines (*p* < 0.05; Fig. 1). Data in Table 2 summarises the responses of consultant and non-consultant trainee neurologists in terms of their knowledge of cannabis-based medicines. None of the study participants reported having no knowledge of cannabinoid medicines, with one consultant and six trainees rating their knowledge as poor. The majority of study participants (*n* = 9 consultants and *n* = 6 trainees; 44% of the overall study cohort) indicated that their knowledge of cannabis-based medicines is average, with only three study participants, consisting only of consultants, rating their knowledge as excellent.

**Table 2:** Participant knowledge of cannabinoid medicines

Level of knowledge	Consultant (*n*)	Trainee (*n*)	Total (*n*)
No knowledge	0	0	0
Poor	1	6	7
Average	9	6	15
Good	5	4	9
Excellent	3	0	3

Study participants rated their knowledge of cannabis-based medicines using a 5-point Likert scale

### Source of information used by neurologists for awareness of the cannabis access programmes in Ireland

**Fig. 2:**
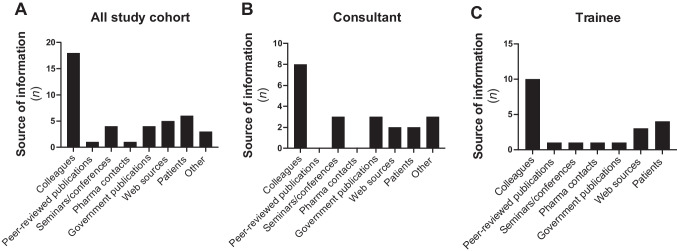
The majority of neurologists most commonly sourced information on the medicinal cannabinoid access programmes in Ireland from colleagues. Participants were asked to indicate how they have predominantly become aware of the medicinal cannabinoid access programmes in Ireland. Data presented as the number of neurologists. Participants could choose more than one option

Given that clinicians report a gap in their knowledge in terms of cannabis-based medicines, in addition to a lack of confidence in such medicines [17, 18], we next set out to determine the sources of information via which study participants became aware of medicinal cannabinoid access programmes in Ireland. Data presented in Fig. 2A indicate that the vast majority of responders most commonly sourced information on access programmes from colleagues (*n* = 18), reporting this as their primary source. Both consultant neurologists (Fig. 2B) and non-consultant trainee neurologists (Fig. 2C) most commonly sourced information on access programmes from colleagues. Participants could choose more than one option, with patients (*n* = 6) and web sources (*n* = 5) indicated as the next most common sources of information used by all neurologists included in this study for awareness of medicinal access programmes in Ireland. Although information regarding the HPRA review of medical cannabis [13] and the MCAP [14] are available via the Department of Health publications, overall, only four (12%) study participants indicated that they have used government publications as a source of information for awareness of the medicinal cannabis access programmes in Ireland.

### The perception and views of neurologists’ of cannabis-based medicines

Study participants were asked to report their perceptions of cannabinoid-based medicines on a 10-point Likert scale (1 = “strongly disagree”; 10 = “strongly agree”), with the data presented in Table 3. Overall, both consultant and non-consultant trainee neurologists indicated that educational programmes on the cannabinoid system and cannabinoid-based medicines are required (mean 10-point Likert score for the full study cohort, 8.53). Neurologists also considered that there is evidence for the use of cannabinoid-based medicines in certain conditions and that they are interested in the use of cannabinoid-based medicines in their practice (mean 10-point Likert scores for the full study cohort, 7.44 and 7.47, respectively) (Table 3). Finally, neurologists included in this study were more divided regarding the clarity of the process of accessing cannabinoid-based medicines. In support of this, the participants did not agree that the process to prescribe/access cannabis-based medications is clear (mean 10-point Likert score for the full study cohort, 4.18) (Table 3). Overall, similar Likert scores were reported by consultant and non-consultant trainee neurologists in terms of their perceptions of cannabinoid-based medicines (Table 3).

**Table 3:** Neurologist’s perspectives on cannabinoid-based medicines

Question	All study cohort			Consultant			Trainee		
	Mean	SD	*n*	Mean	SD	*n*	Mean	SD	*n*
Educational programmes on the cannabinoid system and cannabis-based medicines are required	8.53	2.30	34	9.00	1.41	18	8.00	2.97	16
There is an evidence base to support the use of cannabinoid-based medicines for symptom management in certain conditions	7.44	2.46	34	7.61	2.48	18	7.25	2.52	16
I am interested in the use of pharmaceutical-grade cannabinoid medicines in medical practice	7.47	2.62	34	7.06	2.69	18	7.94	2.54	16
The process to prescribe/access pharmaceutical-grade cannabis-based medications is clear	4.18	2.68	34	4.44	2.77	18	3.88	2.63	16
The process to prescribe/access pharmaceutical-grade cannabis-based medications is unclear	6.00	3.02	33	5.53	3.36	17	6.50	2.63	16

Neurologists’ responses on a 10-point Likert scale (1 = strongly disagree, 10 = strongly agree); mean, standard deviation (± SD) are listed

We next determined the views and awareness of neurologists regarding the access programmes in Ireland. Overall, the majority (74%) of neurologists surveyed in this study indicated that they are aware of the MCAP in Ireland, while just over half (53%) of the full study cohort indicated that they are aware of the Ministerial licence scheme to access medicinal cannabinoid products (Table 4). It is noteworthy that when data from consultants were compared to data from trainees, consultants reported an increased awareness of both the MCAP (83% versus 63%) and Ministerial licence scheme (72% versus 31%) (Table 4). Interestingly, despite evidence that clinicians report a gap in their knowledge in terms of cannabis-based medicines [17, 18], there was universal agreement from all neurologists in our study that they are aware of the endocannabinoid system (85%), with consultants reporting more awareness of the system, when compared to trainees (94% versus 75%) (Table 4). In terms of neurologists’ views on whether the application procedure via the MCAP and Ministerial licence scheme to access medicinal cannabinoids in Ireland is appropriate, 29% of all neurologists surveyed answered “yes”, with 32% indicating “no” (29% of study participants did not answer this questions) (Table 4). When “other” was selected by 9% of all study participants, neurologists completed open-ended questions to determine their views on the application procedures. Quotations from the neurologists are indicated in Table 4, and overall, these indicate that participants describe a lack of knowledge/familiarity with the process.

**Table 4:** Neurologists’ awareness and views of cannabinoids and cannabinoid-based medicines

Question	All study cohort		Consultant		Trainee	
	Yes, *n* (%)	No, *n* (%)	Yes, *n* (%)	No, *n* (%)	Yes, *n* (%)	No, *n* (%)
Are you aware of the MCAP in Ireland?	25 (73.5)	9 (26.5)	15 (83.3)	3 (16.7)	10 (62.5)	6 (37.5)
Are you aware of the Ministerial licensing system for access to medical cannabinoid products?	18 (52.9)	16 (47.1)	13 (72.2)	5 (27.8)	5 (31.3)	11 (68.7)
Are you aware of the endogenous cannabinoid (endocannabinoid) system?^a^	29 (85.3)	4 (11.8)	17 (94.4)	0 (0)	12 (75.0)	4 (25.0)
Have you received anecdotal evidence from your patients in terms of the benefit of cannabis and(or) cannabis-based medicines for their symptoms?	29 (85.3)	5 (14.7)	15 (83.3)	3 (16.7)	14 (87.5)	2 (12.5)
Do you think the application procedure via the MCAP/Ministerial licence scheme to access medicinal cannabinoids in Ireland is currently appropriate?^b^	10 (29.4)	11 (32.4)	6 (33.3)	7 (38.9)	4 (25.0)	4 (25.0)
When “other” selected, the following reasons were provided by study participants: • Not enough knowledge of the system to answer • Not fully abreast of the process to comment • Haven’t used it • Not familiar enough to answer this question well • Not sure						

^a^One study participant did not answer this question

^b^Ten study participants did not answer this question. Three participants indicated “other” to this question

### Anecdotal evidence from patients in terms of the benefit of cannabis and(or) cannabis-based medicines

Our findings indicate that some neurologists surveyed in the study have gained information from patients regarding the medicinal access programmes in Ireland (Fig. 2), with evidence elsewhere supporting the therapeutic use of cannabis-based medicine from anecdotal patient experiences [23]. In support of this, 85% of the study cohort indicated that they have received anecdotal evidence from their patients in terms of the benefit of cannabis and(or) cannabis-based medicines for their symptoms (Table 4). Participants were asked to report both the indication and medication that their patients reported as beneficial for their symptoms, and data presented in Table 5 indicate that spasticity in MS (*n* = 19), pain in MS (*n* = 14) and epilepsy (*n* = 14) were the most common indications from anecdotal patient experiences. Other indications included nausea and vomiting, chronic pain and neuropathy. The most common cannabis-based medicines with reported beneficial effects on symptoms from anecdotal patient experiences were Sativex® (*n* = 17), Epidiolex® (*n* = 13) and CannEpil™ (*n* = 7). Other medications with reported beneficial effects on symptoms from anecdotal patient experiences include Cesamet® (*n* = 1) and several products available under the MCAP programme, including Aurora High CBD Oil drops (*n* = 5), Tilray Oral Solution (*n* = 5), Oleo Bedrocan (*n* = 3), Aurora Sedamen Softgels (*n* = 1) and Oleo Bedrobinol (*n* = 1).

**Table 5:** Anecdotal evidence from patients in terms of the benefit of cannabis and(or) cannabis-based medicines for symptoms

Indication	*N*^a^
Spasticity in MS	19
Pain in MS	14
Epilepsy	14
Nausea and vomiting	1
Other*	5
Medication	*n*
Sativex® (Nabiximol)	17
Epidiolex®	13
Marinol (Dronabinol)	0
Cesamet® (Nabilone)	1
Aurora High CBD Oil Drops	5
CannEpil™	7
Tilray oral solution	5
Aurora Sedamen Softgels	1
Oleo Bedrobinol	1
Oleo Bedrocan	3
Other**	6

^*^Chronic pain; neuropathy; seizures

^**^Cannabis; plant marijuana; not specified

^a^Participants may choose more than one response

### Applications made by neurologists to the MCAP and Ministerial licence schemes in Ireland

At present, cannabis-based medicinal products are not currently available directly in the Irish marketplace [9, 12]. In 2019, the MCAP was formally established [14], with this programme operating in parallel to the Ministerial licence route, a route by which the Minister for Health can consider granting a licence to an Irish-registered clinician to access cannabis products for therapeutic use for a patient under their care [24]. We next determined whether study participants have made applications on behalf of their patient(s) to either the MCAP (Table 6) and(or) Ministerial licence scheme (Table 7). Under one-third of neurologists who participated in the study indicated that they have made applications to the MCAP (32%; Table 6) and Ministerial licence scheme (27%; Table 7) to access cannabis-based medicinal products for a patient under their care. Neurologists were asked to report both the medication and symptoms/conditions for which they made an application. In terms of the MCAP, data presented in Table 6 indicate that applications were made to MCAP to access Sativex® (*n* = 5), CannEpil™ (*n* = 5), Tilray Oral Solution (*n* = 2), Epidiolex® (*n* = 1), Cesamet® (*n* = 1) and Oleo Bedrocan (*n* = 1). Spasticity in MS (*n* = 9), epilepsy (*n* = 4) and pain in MS (*n* = 3) were the most common symptoms/conditions for which applications were made to the MCAP (Table 6). For the Ministerial licence route, data presented herein indicate that applications were made via Ministerial licences to access CannEpil™ (*n* = 5), Sativex® (*n* = 3), Oleo Bedrocan (*n* = 2), Cesamet® (*n* = 1), Tilray Oral Solution (*n* = 1) and Oleo Bedrobinol (*n* = 1) (Table 7). Again, spasticity in MS (*n* = 8) was the most common symptoms/conditions for which applications were made to the Ministerial licence scheme, followed by epilepsy (*n* = 3) and pain in MS (*n* = 2) (Table 7).

**Table 6:** Applications made by study participants to the MCAP

Have you made an application to the MCAP on behalf of your patient(s)?	*n*^a^ (%)
Yes	11 (32.4)
No	20 (58.8)
No answer	3 (8.8)
Indication	*n*
Spasticity in MS	9
Pain in MS	3
Epilepsy	4
Nausea and vomiting	0
Other	0
Medication	*n*
Sativex® (Nabiximol)	5
Epidiolex®	1
Marinol (dronabinol)	0
Cesamet® (Nabilone)	1
Aurora High CBD Oil Drops	0
CannEpil™	5
Tilray oral solution	2
Aurora Sedamen Softgels	0
Oleo Bedrobinol	0
Oleo Bedrocan	1
Other*	1

^*^Bedrocan through the old system

^a^Participants may choose more than one response

**Table 7:** Applications made by study participants to the Ministerial licensing scheme

Have you made an application to the Ministerial licensing scheme on behalf of your patient(s)?	*n*^a^(%)
Yes	9 (26.5)
No	20 (58.8)
No answer	5 (14.7)
Indication	*n*
Spasticity in MS	8
Pain in MS	2
Epilepsy	3
Nausea and vomiting	0
Other	0
Medication	*n*
Sativex® (Nabiximol)	3
Epidiolex®	0
Marinol (Dronabinol)	0
Cesamet® (Nabilone)	1
Aurora High CBD Oil Drops	0
CannEpil™	5
Tilray oral solution	1
Aurora Sedamen Softgels	0
Oleo Bedrobinol	1
Oleo Bedrocan	2
Other*	2

^*^Granted authority to prescribe but have yet to prescribe them to an individual patient

^a^Participants may choose more than one response

## Discussion

We conducted an all-Ireland survey to determine the views and perspectives of Irish neurologists regarding the use of cannabis-based medicines in their practice. Data presented herein indicate that neurologists have an average knowledge of cannabinoid-based medicines, acquired predominantly from colleagues. In addition, our findings indicate that the majority of neurologists surveyed are aware of systems to access cannabis-based products for medicinal use in Ireland, and that approximately one-third of neurologists that completed the study have made applications to the MCAP and(or) Ministerial licence scheme to access cannabis-based medications on behalf of their patient(s). Importantly, this study indicates that educational programmes on the cannabinoid system and cannabis-based medicines are warranted.

The findings of the study indicate that consultant neurologists had a significantly greater score than non-consultant trainee neurologists in terms of the opinion of their knowledge of cannabis-based medicines. This difference in knowledge is likely due to a higher level of clinical experience amongst consultants and greater front-line exposure to more complex clinical cases. In addition, a factor in this regard may be that prescription of cannabis-based medicines is restricted to consultant use only, resulting in a lack of real-world experience for non-consultant hospital doctors. In support of this, our findings indicate that consultants, when compared to trainees, reported an increased awareness of both the MCAP (83% versus 63%) and Ministerial licence scheme (72% versus 31%). The majority (77%) of survey participant’s reported that their knowledge ranged from average to excellent. Clinical guidelines for the medical use of cannabis have been available in Ireland via Government sources since 2020 [12], and the availability of such guidelines may reflect such data. This is somewhat in contrast to findings elsewhere reporting physicians’ experiences and attitudes towards medical cannabis. Indeed, data from Rønne et al. (2021) report that clinicians generally experience a lack of knowledge regarding the clinical effects of medical cannabis, including beneficial and adverse effects [25].

The current regulatory framework surrounding the use and access of cannabis-based medicinal products in Ireland is complex. Our findings indicate that approximately one-third of neurologists included in the study have made an application to the medical cannabis access programme on behalf of a patient. This is consistent with data indicating a perceived lack of evidence-based knowledge amongst clinicians [25]. It is likely that the complexity of regulatory framework in Ireland presents an obstacle to the engagement of clinicians in the access programmes. This may be further compounded by the number of different formulations available and the lack of clarity regarding drug doses. Indeed, authorised cannabis-based medicines in Ireland, and products accessed via the Ministerial licence scheme and MCAP, include products that are administered via oromucosal spray, oils, tablets and via inhalation [14]. With significant and continued policy changes on a country-by-country basis, there is similar regulatory complexity in a European context [26]. Indeed, regulations governing access to cannabis products vary by country [27]. In general, cannabinoid-based medicines, particularly Sativex®, Cesamet® and Marinol®, are authorised in several countries in Europe [26, 28]. As an example, a recent German study indicates that Sativex® is authorised in Germany for the management of spasticity in MS, while Cesamet® is authorised on prescription for chemotherapy-associated nausea and emesis. Marinol® is also available for no-label use in Germany [29]. As a further example, in the UK, cannabis-based medicinal products were re-scheduled by the Home Office in 2018, which facilitated a pathway to prescribing cannabis-based medicines by consultant physicians when conventional treatments have not met treatment targets [30]. Approximately 32,000 patients had received prescriptions for cannabis-based medicinal products in the UK by the end of 2022, predominantly for chronic pain, anxiety and fibromyalgia [31, 32]. Twelve percent of our study cohort was based in the UK, and although our study is statistically underpowered to stratify our findings based on practice location, it is likely that regulatory differences based on geographical location may impact prescription numbers to patients in Ireland.

Our research examined the sources of information utilised by neurologists to learn about cannabinoid-based medicines. We found that “colleagues” emerged as the most common source of information. This is in contrast to data elsewhere indicating that neurologists predominantly access medical literature to formulate their opinions and beliefs regarding cannabis [20, 22]. Indeed, just one neurologist (a trainee) in our study indicated that they access peer-reviewed publications to access information on access programmes. However, it is noteworthy that the use of cannabis and its derivatives for therapeutic purposes is an emerging topic in modern medicine, and it is reasonable to suggest that there may be a lack of peer-reviewed research literature in the context of the Irish healthcare system. Furthermore, study participants indicated that they obtained information from secondary sources, such as websites. Web sources were the next most common source of information by which neurologists accessed information on medicinal cannabis access programmes. This suggests that there is a gap in knowledge amongst neurologists in Ireland regarding cannabinoid-based medicines, and this is supported by 10-point Likert scores (10 = strongly agree and 1 = strongly disagree) regarding the question “Educational programmes on the cannabinoid system and cannabis-based medicines are required” (mean Likert, 8.53; Table 3). Open-ended responses included statements such as “This area needs more awareness among clinicians” and “medical schools do not teach about cannabis dosing/side effects etc. then suddenly we are being asked to prescribe and take responsibility for it with no teaching/training”.

Our findings regarding the need for formal professional training and educational programmes on cannabis and cannabis-based therapeutics for clinicians are supported by data from many studies elsewhere in jurisdictions such as Canada, USA, Israel and Norway [16, 17, 20–22, 33–36]. Indeed, data indicate a need for education to inform clinicians on safety issues [21], with findings from our study and elsewhere indicating that training about medicinal cannabis should be incorporated into medical school curricula [20, 35]. As far as we are aware, there is an absence of formal educational and training programmes on the endocannabinoid system, and cannabis-based medicines, in the Irish healthcare system, and this is likely to impact engagement with access programmes and the number of prescriptions given for medicinal cannabinoid products in Ireland.

Data presented herein indicate that there is a general consensus amongst neurologists that there is sufficient evidence base to support the use of cannabinoid-based medicines for symptom management in certain conditions and that overall, neurologists are interested in the use of cannabinoid medicines in medical practice. This is consistent with studies in other clinical cohorts indicating that the majority of study participants are supportive of incorporating the use of cannabinoids into clinical practice for treatment/symptom management in certain conditions [33, 34, 36]. In Ireland, consultants play a key role in access to cannabis-based medicinal products, and specifically, neurologists are often the patients’ first contact point in the healthcare system to coordinate their treatment given that MS-associated spasticity and severe, treatment-resistant epilepsy are indications recommended under the HPRA review of medical cannabis [13].

The views of study participants regarding the appropriateness of the access programmes to prescribe cannabis-based products were mixed, suggesting that the study cohort is uncertain about navigating the current systems in place. Feedback reported in Table 4 included “Not enough knowledge of the system to answer” and “Not fully abreast of the process to comment”. The regulatory and legal frameworks in Ireland regarding the use and access to cannabis-based medicinal products in Ireland are complex, with the existence of regulatory hurdles and potential uncertainty regarding clinical guidelines. In other jurisdictions, systems are in place to monitor safety and outcomes when prescribing cannabis-based products [37]. It is reasonable to suggest that the development of a national monitoring system (to monitor safety, side effects, adverse events, positive outcomes) may alter the views of clinicians in Ireland regarding the appropriateness of the MCAP and Ministerial licence scheme.

Importantly, at the end of the survey, neurologists completed open-ended responses. Quotations from the study participants are presented in Table 8, with feedback indicating “I think it would be beneficial if a senior SpR/Fellow could also prescribe to take burden of workload off consultants”. This suggests that time constraints, in terms of processing applications to fill prescriptions, may be an important consideration and suggests that a more streamlined process may negate delays in the process and improve engagement.

**Table 8:** Open-ended responses from study participants

Comments
•There should be a national position statement that should be synthesized into a patient information
•This area needs more awareness among clinicians
•System is a mess and needs reorganization and consolidation
•Using medicinal cannabis for about two years at least via this government-based scheme
•Wondering why you haven’t asked regarding how one is finding it working in your patients
•It strikes me as being crude medicine
•It would be easier all around if it was just legal in my opinion
•In N. Ireland we require cost per case approval from the Dept of Health and must meet NICE guidance for Sativex for spasticity in MS
•I think it would be beneficial if a senior SpR/Fellow could also prescribe to take burden of workload off consultants
•Great medication for MS, 12/13 patients doing great
•Need it licenced for pain
•The whole system is a mess, medical schools do not teach about cannabis dosing/side effects etc. then suddenly we are being asked to prescribe and rake responsibility for it with no teaching/training
•I usually go with what the pharmacy in Netherland recommends or how Sativex is prescribed in UK but dosages in Ireland do not allow same maximum dose as UK much lower

### Limitations of the study

The main limitation of this study is the low sample size, although the response rate was in line with studies elsewhere [16, 21, 22, 33]. It is noteworthy that the number of neurologists that completed the study in full reflects the limited size of the Irish neurologist community. The study participants may have been motivated to complete the study based on strong opinions on cannabis use per se, and the prescription of cannabis-based medicines. In addition, the study does not capture the perspectives of other consultants and healthcare providers (e.g. nurse practitioners). This was beyond the scope of the current study and will be the focus of future studies from our group. Finally, neurologists completed open-ended responses to allow study participants to give further views on the study, with quotations from the study participants presented in Table 8. Feedback indicated “why you haven’t asked regarding how one is finding it working in your patients”. Such responses regarding patient feedback views and perspectives on cannabis-based medicines in Ireland will also be a focus of future studies. Despite this, the current study is the first all-Ireland study to determine the perspectives of neurologists on cannabis-based medicines.

## Conclusions

Neurologists are at the forefront of prescribing cannabis-based medicines in Ireland for a restricted set of conditions. The aim of this study was to conduct the first national study in Ireland to determine the views and perspectives of neurologists regarding cannabis-based medicines. Most of the responders in this study indicated that they have average knowledge of cannabis-based medicines, with an appetite for training and formal educational programmes identified. The findings indicate that neurologists are interested in the use of cannabinoid-based medicines. The results point to the need for further education and clarity on guidelines regarding the process for accessing cannabis-based medicines in Ireland, with approximately one-third of neurologists that completed the study indicating that they have made an application to the medical cannabis access programme on behalf of a patient.

## Data Availability

The data may be available upon reasonable request. Some data may not be made available because of privacy or ethical restrictions.
